# Sirolimus and metformin synergistically inhibit hepatocellular carcinoma cell proliferation and improve long-term survival in patients with HCC related to hepatitis B virus induced cirrhosis after liver transplantation

**DOI:** 10.18632/oncotarget.11591

**Published:** 2016-08-25

**Authors:** Chuan Shen, Chenghong Peng, Baiyong Shen, Zhecheng Zhu, Ning Xu, Tao Li, Junjie Xie

**Affiliations:** ^1^ Department of General Surgery, Ruijin Hospital, Shanghai Jiao Tong University School of Medicine, Shanghai, China

**Keywords:** rapamycin, metformin, mTOR, liver transplantation, HCC

## Abstract

Immunosuppressive agents used postoperatively after liver transplantation (LT) for hepatocellular carcinoma (HCC) favor recurrence and metastasis. Therefore, new effective immunosuppressants are needed. This retrospective study assessed combined sirolimus and metformin on survival of HCC patients after LT. In 2001-2013, 133 HCC patients with LT were divided into four groups: sirolimus and metformin combination (Sir+Met), sirolimus monotherapy (Sir), other immunosuppressants in diabetes mellitus (DM) patients without metformin (No Sir with DM), and other immunosuppressants in patients without DM (No Sir without DM). Kaplan-Meier and Log-rank tests were used to assess survival. Cell proliferation and tumor xenograft assays were performed to disclose the mechanisms underlying the sirolimus and metformin effects. The Sir+Met group showed significantly prolonged survival compared to the other groups. The most significant cytotoxicity was seen in the Sir+Met group, with significantly decreased levels of phosphorylated PI3K, AKT, AMPK, mTOR, 4EBP1 and S6K, compared with the other groups. In agreement, Sir+Met had the highest suppressive effect on tumor growth among all groups (P<0.01). In summary, Sir+Met treatment significantly prolonged survival, likely by suppressing cell proliferation. Therefore, this combination could represent a potential routine-regimen for patients post LT.

## INTRODUCTION

Hepatocellular carcinoma (HCC) is the second most common cause of cancer death worldwide, and half of the estimated 782,000 new cases occurred in China in 2012; HCC is the fifth most common cancer in men (554,000 cases, 7.5% of total cases) and the ninth in women (228,000 cases, 3.4%) [1]. Liver cancer prognosis is very poor and treatments with curative intent include liver transplantation (LT), liver resection (LR), and radiofrequency ablation (RFA) [2]. LT seems to be the most effective method, but requires postoperative immunosuppressive agents, which potentially promote recurrence and metastasis [3].

Calcineurin inhibitors (CNIs) are the most commonly used immunosuppressants for solid organ transplantation, including LT. Alone or in combination with other immunosuppressive drugs, CNIs achieve a significant reduction in acute rejection rates, increasing graft and patient survival [3, 4]. However, their side effects are also remarkable and include cardiovascular complications, nephrotoxicity, neurotoxicity, diabetes, HCC recurrence and development of *de novo* tumors [3]. It is possible that hematopoietic stem cells, that may reside in adult liver, cause hepato-carcinogenesis [5]. Mammalian target of rapamycin (mTOR) is a serine-threonine kinase that regulates cell growth, proliferation, survival, metabolism, and angiogenesis via the phosphatidylinositol 3-kinase/protein kinase B (PI3K/AKT) signaling pathway [6]. mTOR occurs in two multiprotein complexes (mTORC1 and mTORC2), of which mTORC1 is bound by the macrolide antibiotic rapamycin (sirolimus) and its analogs everolimus, temsirolimus, and ridaforolimus [7, 8]. Because of their relative lack of nephrotoxicity, mTOR inhibitors (mTORi), including sirolimus and everolimus, are considered promising alternatives for CNIs. Indeed, blocking mTOR with sirolimus (an immunosuppressive agent widely used post LT) and first generation mTORi, has shown promising reduction of HCC tumor growth in preclinical models [9, 10] and in some recent clinical trials [11, 12].

The PI3K/AKT/mTOR pathway can be negatively regulated by phosphatase and tensin homolog (PTEN), SH2-containing inositol phosphatase-1 (SHIP1) and PITenins (PITs) [13, 14]. The PI3K-AKT and Ras-Raf-Mek-Erk signaling pathways activate mTORC1 by phosphorylating and thereby inhibiting the tumor suppressors tuberous sclerosis 1 and 2 (TSC1-TSC2) complex [15, 16]. Inhibitors of Ras-Raf-Mek-Erk signaling are considered to be potential agents for HCC treatment [17]. The mechanism of mTORC2 is less well understood. It is insensitive to nutrients but responds to growth factors such as insulin in association with ribosomes [18].

Metformin (1, 1-dimethylbuguanide hydrochloride), an antidiabetic agent of the biguanide class, is widely used to treat type II diabetes mellitus (DM). It improves insulin sensitivity in diabetics, affecting many tissues such as the liver, skeletal muscle, adipose tissue, endometrium and ovaries [19]. Although metformin has been used for more than 40 years, its mechanism of action is not fully understood [20]. DM increases the risk of HCC three-fold [21]. and in recent years, anti-DM drugs such as metformin are emerging as potential anticancer agents [22–25] including in HCC [21, 26]. Metformin probably influences mitochondria in cancer cells to reduce tumorigenesis [27, 28], and inhibits both insulin-like growth factor (IGF) signaling and mTOR; it also modulates AMP kinase (AMPK), represses the AKT/mTOR pathway, eliminates cancer stem cells (CSCs) and inhibits tumor growth [29]. Both mTOR and AMPK control the cellular energy supply through the ATP to AMP ratio and regulate key aspects of cell growth. Interestingly, insulin/IGF receptor signaling is regulated via the phosphatidylinositol 3-kinase/AKT/mTOR signaling pathway [30]. It has been shown that administration of metformin inhibits human pancreatic adenocarcinoma PANC-1 and MiaPaCa-2 tumor xenografts *in vivo* in a dose-dependent manner [31]. Similar findings were obtained in HCC: metformin suppresses hepatocellular carcinoma cell growth through induction of cell cycle G1/G0 phase arrest, p21CIP and p27KIP expression, and downregulation of cyclin D1 *in vitro* and *in vivo* [32].

These findings indicate that both sirolimus and metformin act on the PI3K/AKT/mTOR pathway. We hypothesized that the synergistic effect of these two molecules would result in effective inhibition of tumorigenicity in HCC. Indeed, clinical trials suggest that mTOR inhibition by everolimus alone has only moderate antitumor efficacy in patients with HCC [33, 34], which may be due to feedback activation of AKT after mTOR inhibition [35, 36]. This feedback can be overcome with improved antitumor and antiangiogenic effects by co-treatment strategies such as, AZD6244/sirolimus [37], sirolimus/bevacizumab [38] and mTOR inhibitors and OSI-906, a blocker of IGF1R/IR combination therapy [39]. Based on these findings, this study aimed to assess the combined therapy of sirolimus and metformin in orthotopic LT. We found that sirolimus and metformin treatment significantly prolonged patient survival, likely via suppression of cell proliferation, indicating that this combination should be considered as a standard prescription after liver transplantation.

## RESULTS

### Clinical study

**Figure 1:**
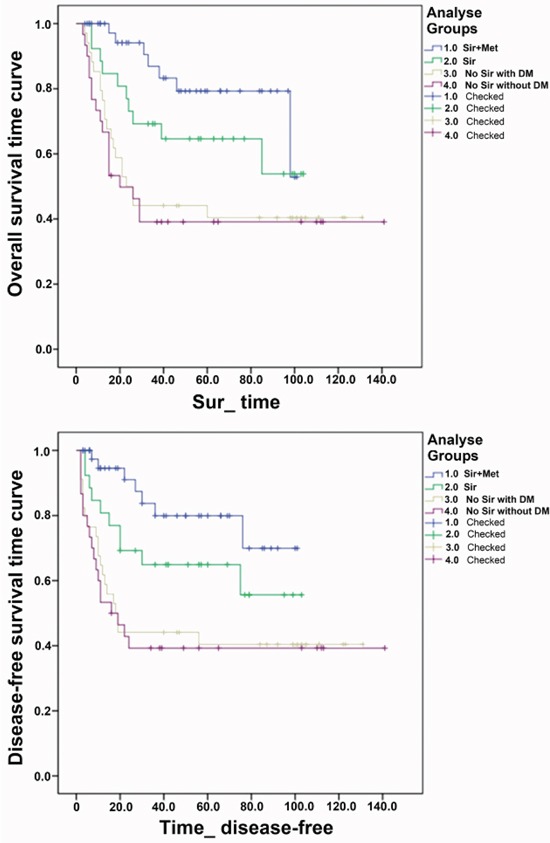
Overall survival time curve and disease-free survival time curve in the four groups: Group 1.0: the sirolimus and metformin combination (Sir+Met); Group 2.0: sirolimus monotherapy (Sir); Group 3.0: No Sir with DM; Group 4.0: No Sir without DM

Table 1 shows the baseline demographic and clinical characteristics of the study population. The patients included 126 males and 7 females averaging 49.2 years old. They had HCC related to HBV infection; HCC stage on explant pathology was within the Milan criteria in 83 patients (62.4%), with microvascular invasion in 47 patients (35.3%). Patients were divided into four groups according to baseline immunosuppression, and the sirolimus and metformin (Sir+Met) combination, sirolimus monotherapy (Sir), No Sir with DM, and No Sir without DM included 43 (32.3%), 26 (19.5%), 34 (25.6%), and 30 (22.6%) patients, respectively. Interestingly, survival in the Sir+Met group was 85.780±5.257 months, significantly longer compared with values obtained with the other groups, including No Sir with DM (63.184±9.795) and No Sir without DM (63.178±11.581) groups (Tables 1 and 2). Log-rank test revealed no significant differences between other group pairs (Figure 1 and Table 2).

**Table 1:** Main baseline demographic and clinical characteristics of the study population (n=133)

	Sir+Met, n (%)	Sir, n (%)	No Sir with DM, n (%)	No Sir without DM, n (%)
Patient(M/F)	41/2	25/1	32/2	28/2
Age, y	49.3±7.1	46.6±9.1	52.0±6.6	48.1±9.9
Primary LT, n(%)	43(100)	26(100)	34(100)	30(100)
Indication to LT by HBV, n (%)	43(100)	26(100)	34(100)	30(100)
HCC stage (within Milan) on explant, n (%)	30	18	20	15
Microvascular invasion on explant pathology, n (%)	15	11	20	12
LT with DM, n (%)	43(100)	2(7.69)	34(100)	0(0)
Survival time (M)	85.780±5.257	71.915±8.058	63.184±9.795	63.178±11.581

**Table 2:** Total 133 patients were divided into four groups according to baseline immunosupression

	Analysis Groups	Sir+Met		Sir		No Sir with DM		No Sir without DM	
		χ2	p	χ2	p	χ2	p	χ2	p
Log Rank (Mantel-Cox)	Sir+Met			2.110	.146	11.323	.001	13.864	<.001
	Sir	2.110	.146			2.638	.104	3.655	.056
	No Sir with DM	11.323	.001	2.638	.104			.152	.696
	No Sir without DM	13.864	<.001	3.655	.056	.152	.696		

The mean overall survival for the Sir+Met group was 85.65 months (SE 5.37, 95% CI: 75.13, 96.17), which was longer that seen in all of the other groups (Table 3).

**Table 3:** The mean of overall survival in the four groups

Groups	Mean (months) of OS		95%confidence interval	
	estimated value	Standard error	lower limit	upper limit
Sir+Met	85.65	5.37	75.13	96.17
Sir	63.0	7.9	47.82	78.35
No Sir with DM	53.50	8.40	37.04	69.96
No Sir without DM	56.52	9.60	37.70	75.34

The toxicity and side effects of the four different treatments showed similar rates between groups (Table 4). In the Sir+Met group the most common side-effects were hypercholesterolemia, anemia, rash, thrombopenia, and diarrhea and these were also seen in the other groups.

**Table 4:** The toxicity and side effects encountered in the four groups of Sir+Met, Sir, No Sir with DM and No Sir without DM

	Sir+Met	Sir	No Sir with DM	No Sir without DM
Hypercholesterolemia	4	4	3	2
Rash	3	2	1	0
Thrombopenia	3	4	3	4
Anemia	4	4	5	3
Hyperlipidemia	2	1	3	2
Hypertension	2	2	5	4
Acne	1	2	0	0
Arthralgia	0	1	1	0
Nausea and vomiting	0	0	1	0
Diarrhea	3	2	1	2
Allergy	0	0	0	0
Vertigo	1	1	0	1
Fatigue	0	0	1	0
Opportunistic infection	1	1	3	1
Lactic acidosis	0	0	1	0
Renal toxicity	0	0	3	2

### Rapamycin and metformin in combination suppressed HCC cell proliferation

**Figure 2:**
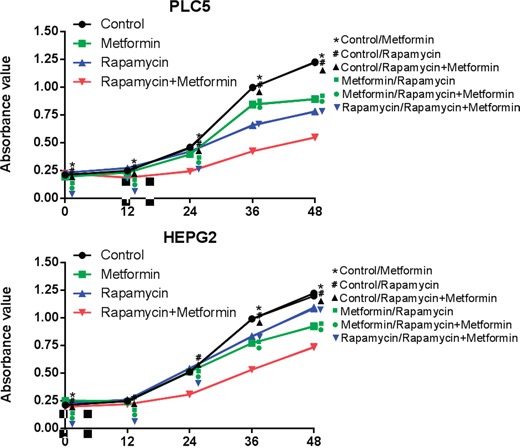
Effects of combined treatment of rapamycin and metformin on cell proliferation Inhibition of cell proliferation was observed in cells treated with either rapamycin or metformin. The most significant inhibition of cell growth was observed in cells treated with both rapamycin and metformin. *P<0.05 between Control and Metformin group; #P<0.05 between Control and Rapamycin group; ▲ P<0.05 between Control and Rapamycin+Metformin group; ■ P<0.05 between Rapamycin and Metformin group; ● P<0.05 between Metformin and Metformin+Rapamycin group; ▼ P<0.05 between Rapamycin and Metformin+Rapamycin group.

Cell proliferation was assessed following the treatment with rapamycin and/or metformin of HepG2 and PLC/PRF/5 cells. Cell viability decreased at 12 h in the rapamycin and metformin in combination group but remained similar in the rapamycin and metformin groups until 24 h. As shown in Figure 2, rapamycin and metformin monotherapies did not significantly inhibit cell proliferation compared with the control group in both HepG2 and PLC/PRF/5 cells (P<0.01). Interestingly, rapamycin and metformin in combination significantly inhibited cell proliferation of both cell lines compared with either rapamycin or metformin groups (P<0.01) (Figure 2).

### Rapamycin and metformin inhibit the PI3K/AKT pathway

**Figure 3:**
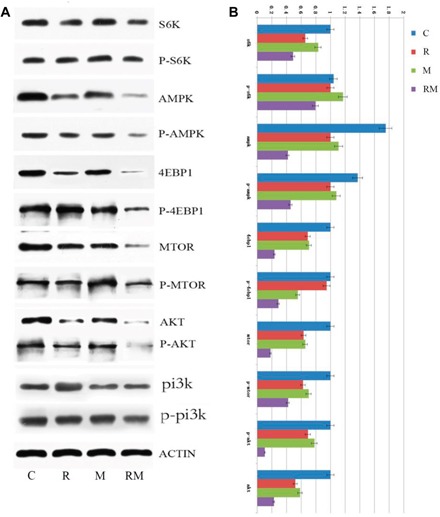
Effects of Rapamycin and metformin on the expression of proteins involved in the PI3K/AKT pathway C: control; R: rapamycin; M: metformin; RM: combined rapamycin and metformin. Rapamycin and metformin combination caused a significant decrease in phosphorylated PI3K (P-PI3K), P-AKT, AKT, P-AMPK, AMPK, P-mTOR, mTOR, P-4EBP1, 4EBP1 and S6K levels.

As shown in Figure 3, combined treatment with rapamycin and metformin resulted in significantly decreased phosphorylated PI3K, AKT, AMPK, mTOR, 4EBP1 and S6K protein levels. Moreover, the rapamycin and metformin combination dramatically decreased the protein levels of PI3K, AKT, AMPK, mTOR, 4EBP1 and S6K, indicating that the rapamycin and metformin combination inhibited PI3K/AKT, AMPK, and the mTOR pathway.

### Rapamycin and metformin in combination suppressed tumor growth *in vivo*

**Figure 4:**
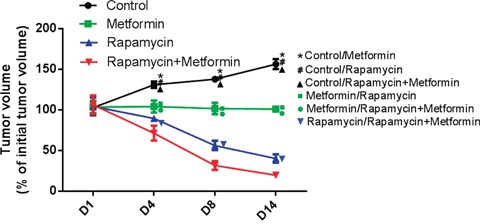
Effects of rapamycin and metformin on tumor growth *in vivo* rapamycin or metformin alone suppressed tumor growth *in vivo*. The rapamycin and metformin combination had the most pronounced inhibitory effect compared with monotherapies. *P<0.05 between Control and Metformin group; #P<0.05 between Control and Rapamycin group; ▲ P<0.05 between Control and Rapamycin+Metformin group; ■ P<0.05 between Rapamycin and Metformin group; ● P<0.05 between Metformin and Metformin+Rapamycin group; ▼ P<0.05 between Rapamycin and Metformin+Rapamycin group.

To further assess the effects of the rapamycin and metformin combination, 2.5×10^7^ PLC/PRF/5 cells were injected into the right flanks of NOG mice for tumor generation, followed by treatment with the test articles as described above. At 8 days, tumor sizes were significantly reduced by rapamycin and metformin monotherapies compared with the control group (P<0.01). However, treatment with the rapamycin and metformin combination resulted in a greater decrease compared with either single drug (P<0.01) (Figure 4).

## DISCUSSION

This retrospective study demonstrated that patients treated with the sirolimus/metformin combination had significantly longer survival than the control group among HCC patients after LT. In accordance, the rapamycin/metformin combination dramatically reduced tumor growth *in vitro* and in a mouse xenograft model, likely via inhibition of the PI3K/AKT/mTOR pathway.

Patients treated with the sirolimus/metformin combination had significantly longer survival than the other groups, in agreement with studies assessing rapamycin monotherapy. Cholongitas E et al. [11] found that patients on calcineurin inhibitors (CNIs), compared with those receiving mTORi, had higher recurrence rates of HCC within Milan criteria before LT and similar rates outside Milan criteria. While LT patients with HCC receiving sirolimus showed no improvement in long-term recurrence free survival but this was improved at 3-5 years alongside OS [12]. No data were available regarding the association of HCC recurrence and the mTORi (sirolimus/everolimus) dosage or blood levels, and no difference was observed in recurrence rates between patients who received steroids for less than 3 months and those treated for a longer period after LT.

Due to the feedback activation of AKT after mTOR inhibition, sole use of mTORi everolimus shows only moderate antitumor efficacy [40]. Interestingly, vertical blockade of the IGFR-PI3K/AKT/mTOR signaling pathway can synergistically improve treatment efficacy in HCC [36]. Recently, meta-analysis determined that the antidiabetic drug metformin reduces the incidence of many cancers and their resulting mortality [40]. For pancreatic cancer (PC), the beneficial effects of metformin were observed in all disease stages but became statistically significant only in patients with non-metastatic disease, suggesting that metformin can be used as a chemopreventive agent in high-risk individuals with early stage PC. On the other hand, metformin has known effects on metabolism and weight control, and can be used in conjunction with a lifestyle intervention program in obese people to achieve weight loss and improve insulin sensitivity [41, 42]. Here, sirolimus and metformin, two widely used medicines that act at different points in the PI3K/AKT/mTOR pathway, were tested synergistically for their inhibitory effects on HCC both *in vivo* and *in vitro*. And we validated our clinical findings that patients that received combined treatment have better prognosis than others receiving monotherapy in the present study. These important indications suggest that the sirolimus/metformin combination could be prescribed to all recipients after LT. Indeed, the combination therapy caused no more toxicity in patients post LT in our retrospective study. Finally, the survival of the patients with sirolimus treatment including both the Sir group and the Sir+Met group was significantly longer compared with the control groups, which suggests that sirolimus improves the prognosis of patients with HCC [11, 12]. But DM, by its complex nature, is an important confounder in this setting.

This study is not without limitations. The clinical study was retrospective, with no strict randomization or blinding. In addition, this was a single center study with a relatively small sample size, and we attempted to eliminate the possible bias due to the different time of drug use as well as distinct treatment durations. However, in the sirolimus/metformin combination group, the addition point of metformin was not strictly unified and metformin was not discontinued even when the patient was a temporary diabetic. Patients with post-LT recurrences were treated with TACE, radio frequency, resection of the metastases (if possible). These treatments would be another potential confounder for the study. Therefore, the efficacy and long-term safety of the sirolimus/metformin combination must be evaluated in well-designed prospective multicenter trials, with large sample sizes.

In summary, the sirolimus/metformin combination effectively prolonged survival in LT patients, and suppressed tumor growth *in vivo* and *in vitro*. The synergistic effects of sirolimus and metformin indicate that these drugs could be routinely prescribed for patients post LT.

## MATERIALS AND METHODS

This was a retrospective study of 283 patients that underwent LT between January 2001 and December 2013 at Shanghai Ruijin Hospital affiliated to Shanghai Jiaotong University School of Medicine. Patients were mostly male (87.3%, 247) aged 48.7±9.54 years. The eligible patients were adult (>18 years old) LT recipients for HCC related to hepatitis B virus induced cirrhosis within and beyond the Milan criteria. A total of 133 patients were enrolled in this study (Table 1) and divided into four groups: 1) sirolimus and metformin combination (Sir+Met); 2) sirolimus monotherapy (Sir); 3) other immunosuppressants in DM patients without metformin (No Sir with DM); 4) other immunosuppressants in patients without DM (No Sir without DM) (Table 2). The primary endpoints were overall survival (OS) and disease-free survival (DFS) in patients. The secondary endpoints were the effects of rapamycin and metformin combination on cell proliferation, tumor growth, and PI3K/AKT, AMPK, mTOR pathway activities. Data were collected at the time of last observation, patient's death, or December 31, 2013.

### Immunosuppression

The dose of sirolimus was 2 mg.D^−1^ (the maximum dose) initially and was reduced by the plasma concentration. Metaformin was initially administered orally at a dosage of 850 mg twice daily or three times a day for patient who was diagnosed as diabetic. The immunosuppressant regiments we studied were postoperative long term maintenance plans, which were administered for three months after LT, when the sequential steroid scheme and monoclonal antibody induced scheme had already been performed.

### Cell culture

The human hepatocellular carcinoma cell lines HepG2 and PLC/PRF/5 were obtained from the Chinese cell line bank, and cultured at 37°C in a humidified environment containing 5% CO_2_, in Dulbecco's modified Eagle medium with Glutamax-1 (HyClone, Logan, UT, USA) supplemented with 10% fetal bovine serum (FBS, Thermo Scientific, USA), 100 U/ml penicillin and 100 g/ml streptomycin.

### Cell viability assay

Cells were treated with either vehicle (control), rapamycin (Sigma, 20 nmol/L, 5μM) [43], metformin (Enzo Life Sciences, 0, 12.5, 25, 50, 100, and 200μM), or rapamycin (Sigma) plus metformin for 12, 24, 36, and 48 h, respectively. One day prior to treatment, cells (1×10^4^) were seeded in 96-well plates. Appropriate amounts of the compounds were then added to achieve the indicated concentrations. Cell growth and proliferation were determined with a Cell Counting Kit-8 (Dojindo, Japan). After addition of 10 μl CCK-8 solution to each well, cells were incubated for 1.5 h, and absorbance was measured on a microplate reader (Spectramax 190, US) at 450 nm.

### Western blotting

Total protein lysates (25–50μg) were separated by sodium dodecyl sulfate-polyacrylamide gel electrophoresis (SDS-PAGE) and transferred onto polyvinylidene difluoride (PVDF) membranes (Millipore, MA, USA). Primary antibodies included anti-P-S6K/S6K (Abcam, UK), P-AMPK/AMPK (Abcam, UK), P- mTOR/mTOR (Abcam, UK), P-4EBP1/4EBP1 (Santacruz, USA), P-AKT (Abcam, UK)/AKT (Novus, USA), P-PI3K/PI3K (Santacruz, USA), and actin (Santacruz, USA) antibodies. After incubation with primary and secondary antibodies, proteins were stained with an enhanced chemiluminescence (ECL) kit (Ab Frontier) and were exposed to X-ray film. Band intensities were quantified using the Image J software (version 1.43; National Institutes of Health, Bethesda, MD, USA). Relative protein expression levels were quantified by normalization to β-ACTIN.

### Tumor xenograft assay

Male NOG mice were maintained under a 12 h light/12 h dark cycle at 22°C with water *ad libitum*, and fed standard laboratory chow. Four-to-six-week-old male NOG mice were used to establish HCC xenografts. All animal experiments were approved by the Institutional Animal Care and Use committee of Shanghai Jiao Tong University. PLC/PRF/5 cells (8 × 10^6^) were injected subcutaneously into the right flanks of NOG mice and allowed to grow for 14 days. Then, mice were randomized into four groups, including (1) vehicle control, (2) rapamycin alone (2 mg/kg, daily oral administration), (3) metformin (250 mg/kg, daily intraperitoneal injection), and (4) rapamycin and metformin combination (same doses, routes, and schedules as with the respective single agents) [44], and treated for 8 days. Tumor dimensions were measured using calipers every other day, and tumor volumes derived as [(length+width)/2]^3^ ×0.5236.

### Statistical analysis

A descriptive approach was used to summarize study characteristics and outcome. Quantitative variables were expressed as mean±standard deviation (SD) and/or median values (range). The median follow-up time was 35.0 months, which ranged from 4.0 to 141.0 months. The Kaplan-Meier method was used to assess survival rate. Log-rank test was used to compare the survival rates among the four groups. Data analysis was carried out with the Statistical Package for Social Sciences software (SPSS 22.0; IBM SPSS, USA). P< 0.05 was considered statistically significant.
